# Avoiding the Removal of Syndesmotic Screws after Distal Tibiofibular Diastasis Repair: A Benefit or a Drawback?

**DOI:** 10.3390/jcm11216412

**Published:** 2022-10-29

**Authors:** Samer Hosin, Dinu Vermesan, Radu Prejbeanu, Dan Crisan, Musab Al-Qatawneh, Daniel Pop, Mihai Mioc, Felix Bratosin, Bogdan Feciche, Kakarla Hemaswini, Marius Liviu Moise, Catalin Dumitru, Vlad Bloanca, Ciprian Nicolae Pilut

**Affiliations:** 1 Department of Orthopedics, “Victor Babes” University of Medicine and Pharmacy Timisoara, 300041 Timisoara, Romania; 2 Methodological and Infectious Diseases Research Center, Department of Infectious Diseases, “Victor Babes” University of Medicine and Pharmacy, 300041 Timisoara, Romania; 3 Department of Urology, Satu-Mare County Emergency Hospital, Strada Ravensburg 2, 440192 Satu-Mare, Romania; 4 Malla Reddy Institute of Medical Sciences, Suraram Main Road 138, Hyderabad 500055, India; 5 Department of Radiology, “Premiere” Hospital—“Regina Maria”, Calea Aradului 113, 300643 Timisoara, Romania; 6 Department of Obstetrics and Gynecology, “Premiere” Hospital—“Regina Maria”, Calea Aradului 113, 300643 Timisoara, Romania; 7 Department of Plastic Surgery, “Victor Babes” University of Medicine and Pharmacy Timisoara, Eftimie Murgu Square 2, 300041 Timisoara, Romania; 8 Multidisciplinary Research Center on Antimicrobial Resistance (MULTI-REZ), Microbiology Department, “Victor Babes” University of Medicine and Pharmacy, Eftimie Murgu Square 2, 300041 Timisoara, Romania

**Keywords:** tibiofibular diastasis, ankle fracture, ankle instability, orthopedic management, foot disorders

## Abstract

There is still no general agreement about the most effective form of syndesmosis fixation with syndesmotic screws for patients affected by ankle fractures that are accompanied by syndesmotic injuries. In the same manner, no consensus has been reached yet on whether or not the tibiofibular syndesmotic screw is more beneficial if removed or not, as well as the exact timing of removal if this proves superiority. The purpose of this research was to verify whether or not removing syndesmotic screws reduces the risk of developing a diastasis and compare outcomes in patients whose syndesmotic screw was or was not removed at all. A retrospective observational study was carried out to cover a period of five years and a computed sample size of almost 300 cases. Patients were included in the current study if their history was positive for ankle fracture with distal tibiofibular diastasis repair with syndesmotic screws. Loss of reduction was more frequent after screw removal (8.5% vs. 2.1%), although the quality of reduction was generally excellent in both groups. The mean AOFAS score was significantly better in patients who had their tibiofibular screw removed (92.6 vs. 88.4), but the tibiofibular clear space and incisura fibularis depth widened more following the second intervention (3.8 mm vs. 3.6 mm, and, respectively, 4.3 vs. 4.1). Lastly, the same patients with tibiofibular screw removal had a significantly higher cost of total interventions and more days of medical leave (21 vs. 15 days on average). It seems that a strong conclusion in favor of removing or not removing syndesmotic screws after distal tibiofibular diastasis repair cannot be given. However, several radiographic findings lean toward the benefit of those patients whose tibiofibular screws were not removed, although mobility was notably better after the screw was removed. Furthermore, treatment expenses are greatly lowered if a subsequent operation for screw removal is avoided, as well as if individuals who have a single surgery take a shorter medical leave.

## 1. Introduction

Fractures of the ankle are one of the most prevalent types of injuries that may involve the lower limb, affecting mostly young men and elderly women [1,2]. These fractures may happen with or without dislocation, considering only around ten percent are caused by trauma [3]. A fracture-dislocation of the ankle is a rare injury produced by severe trauma that includes the bone and soft tissues surrounding the ankle or in any other combination [4,5]. Immediate closed reduction in ankle dislocation and temporary immobilization with an external fixator or bivalved cast to maintain stabilization post-reduction is a crucial step in the initial management of these severe injuries while awaiting definitive synthesis that involves open reduction and internal fixation as the procedure of choice [6,7].

An undamaged distal syndesmotic tibiofibular ligament complex is crucial for the stability and function of the ankle joint [8]. As a result, discomfort and joint wear may result from syndesmotic injuries that do not go through proper management. Therefore, it is crucial to restore a congruent articulating ankle with stable syndesmosis in order to avoid post-traumatic osteoarthritis, while in rare cases, syndesmosis injury may even cause serious limb-threatening complications [9,10]. 

Depending on the categorization of fractures, syndesmosis damage occurs in approximately 15% of ankle fractures and one-fifth of all ankle fractures that may need surgical fixation [11,12,13]. This is because these injuries are linked with altered tibiofibular joint kinematics. In the surgical treatment of syndesmosis injuries, the use of screws as a means of fixation has shown to be the most successful approach. However, there is continuing disagreement about the practice of routinely removing screws, which is often performed to reduce the risk of screw breakage. There are data to show that removing screws is related to higher costs, an increased risk of infection, and an earlier loss of syndesmosis reduction [14,15]. In contrast, more recent investigations using computed tomography (CT) have shown that removing the screws may make it possible for a malreduced syndesmosis to shrink spontaneously [16]. 

As a direct consequence of these contradictory and inconsistent findings, the need for alternate fixation mechanisms has increased. Implantable suture button devices were introduced with the advantage of allowing micromotion during the healing process and potentially eliminating the need for routine hardware removal [17]. This was performed to address the shortcomings of screw fixation, which had been the traditional method of bone fixation [18]. There are accumulating data to suggest that the suture button has equivalent mechanical strength attributes to those of screws, which in turn results in clinical outcomes that are comparable [19]. However, according to a number of studies, the rates of suture button removal after syndesmosis fixation may reach as high as forty percent, due to reasons that comprise hardware irritation, infection, osteomyelitis, and osteolysis [20,21,22]. Moreover, new orthopedics devices and procedures are not widely available, and their costs make them inaccessible.

There have been very few studies that have described early and long-term results and complications of surgically repaired distal tibiofibular diastasis in relationship with the removal of syndesmotic screws [23]. Therefore, the goal of the current study was to report the results from 308 patients who had been surgically treated by open reduction and internal trans-syndesmotic fixation for ankle fracture with dislocation. Assuming that not removing the trans-syndesmotic fixation will not have detrimental effects on the patients, we aimed to observe any related effects or complications.

## 2. Materials and Methods

### 2.1. Study Design and Settings

The current study followed an observational retrospective design where patients were included in the study if their hospital admission occurred between January 2017 and January 2022. The research was carried out at the University Clinic of Orthopedics affiliated with the “Victor Babes” University of Medicine and Pharmacy in Timisoara. The administrative database that was used belonged to the clinic’s inpatient population and comprised both the study population and the features that were considered relevant. The major complaints, demographic information, surgeries performed, and other clinical data were identified from digital and paper records. These types of patient data were protected by privacy legislation and the patients’ agreement that were examined by certified physicians and other approved healthcare workers who were taking part in the present research project.

The orthopedy clinic, as an auxiliary of Timis County Emergency Clinical Hospital “Pius Brinzeu”, operates under the laws of the Local Commission of Ethics that approves Scientific Research that functions in accordance with the following regulations: (1) Article 167 of Law No. 95/2006, Art. 28, Chapter VIII of Order 904/2006; (2) the EU GCP Directives 2005/28/EC; (3) the International Conference on Harmonisation of Technical Requirements for Registration of Pharmaceuticals for Human Use (ICH). On 16 February 2022, the inquiry that is now underway was granted permission to proceed and was assigned the number 38.

### 2.2. Participants and Definitions 

Patients with a history of unimalleolar, bimalleolar, or trimalleolar ankle fractures were considered eligible for inclusion in the current study. They were identified by the International Classification of Diseases (ICD-10) diagnosis codes [24], together with finding talofibular syndesmotic instability that required talofibular trans-syndesmotic screws, according to existing guidelines. In the Danis–Weber classification system, an ankle fracture is assigned to one of three kinds (A, B, and C) depending on the position of the lateral malleolus fracture. This method is one of the most extensively used classification systems, describing that the fracture of the lateral malleolus in type A occurs below the syndesmosis, whereas in type B, it occurs across the syndesmosis, and in type C, it occurs above the syndesmosis. The fractures of type A and type B are considered stable. A fracture of type C is considered unstable and may be treated with talofibular trans-syndesmotic screws that can be removed through a secondary intervention when the surgeon considers, which is at approximately one year after the initial intervention [25,26]. The Lauge–Hansen grading system was also used to classify the various types of fractures in these patients as follows: SER—supination external rotation; SA—supination adduction; PER—pronation external rotation; and PA—pronation abduction [27].

Clinical and radiographic examination criteria were used to evaluate the progress of fracture healing, such as pain at the fracture site while the patient was bearing weight and assessing callus formation or secondary fracture lines. The clinical results were evaluated using the American Orthopedic Foot and Ankle Society Score (AOFAS) at the most recent follow-up, which was carried out in our hospital. This score evaluates the range of motion, stability of the ankle, pain, other symptoms affecting the activities of daily living, ability to practice sports, and quality of life [28]. Radiographic examination of the ankle was performed in order to diagnose post-traumatic osteoarthritis using the Van Dijk grading criteria [29], as follows: (0)—a normal joint or subchondral sclerosis; (I)—the presence of osteophytes without joint space narrowing; (II)—joint space narrowing with or without osteophytes; and (III)—disappearance or deformation of the joint space. 

**Figure 1:**
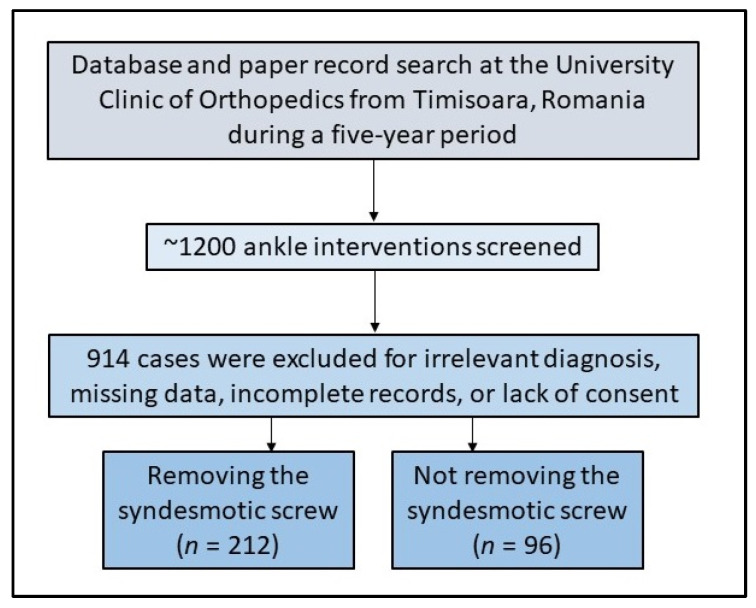
Flowchart displaying cases included in the current study.

Other inclusion criteria were the age of the patient as equal to or older than 18 years of age and the personal approval of the patient for being enrolled in clinical studies with full private records, including imaging studies and photos of the affected limbs. Patients were removed from the research if their medical records were found to be lacking important data and other criteria described in Figure 1. It was determined using a convenience sampling technique that a total of 287 patients would be sufficient for the results to have statistical power. The sample size was calculated for a 95% confidence interval, at a 5% margin of error, and a 1–3% prevalence of ankle fractures in the general population. 

### 2.3. Variables

The variables of interest for the current study comprised: (1) background characteristics—age, sex, body mass index (BMI), area of residence, relationship status, level of income, level of education, occupation, substance-use behavior, chronic comorbidities; (2) intervention features and imaging characteristics of the study participants—soft tissue condition, side of injury, time between injury and definitive surgery, Danis–Weber classification, fracture type, trauma type, outcomes, orthopedic complications; (3) patient follow-up at six months—average time to screw removal, post-operative rehabilitation, AOFAS score, Lauge–Hansen classification, Van Dijk score, range-of-movement (ROM) assessment, quality of reduction; and (4) differences in radiographic changes between the two study groups—mean tibiofibular overlap (TOL), mean tibiofibular clear space (TCS), mean incisura fibularis depth (IFD), tibiofibular overlap change, tibiofibular clear space change, incisura fibularis depth changes.

### 2.4. Statistical Analysis

IBM SPSS version 27.0 (SPSS. Inc., Chicago, IL, USA) and Microsoft Excel (Microsoft Corp., Redmond, WA, USA) were used for statistical analysis. The representation of categorical variables was accomplished by absolute values and the frequencies of those values. A statistical examination of the proportions was carried out with Chi-square and Fisher’s exact tests. A Shapiro–Wilk test was performed to determine the Gaussian distribution of data, and a Student’s *t*-test was carried out to compare the means of Gaussian variables. A level of significance of 0.05 was chosen as the threshold for the alpha value.

## 3. Results

### Patient Characteristics

**Figure 2:**
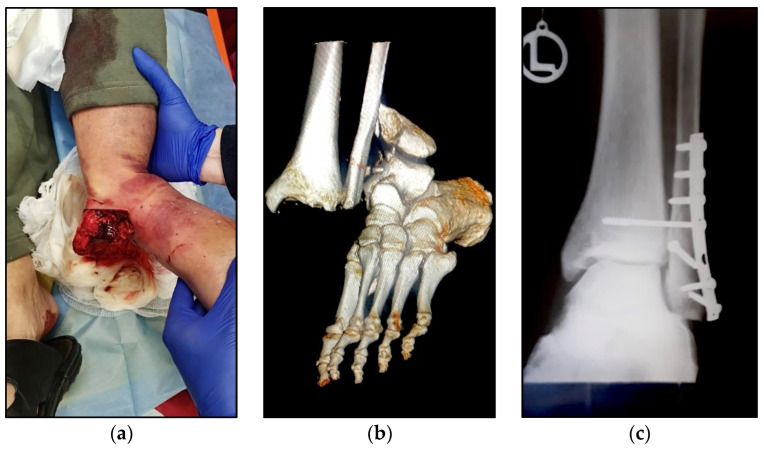
Examples of initial presentation and radiographic findings in severe ankle fractures that require talofibular trans-syndesmotic screws: (**a**) pre-operative aspect; (**b**) pre-operative computed tomography 3D reconstruction; (**c**) post-operative X-ray in frontal view.

A total of 308 patients were identified with a positive history of ankle fracture with distal tibiofibular diastasis repair with syndesmotic screws. In this population, 212 patients underwent a second surgical intervention for syndesmotic screw removal, while the other cases were left without removing the screw. Table 1 shows the comparison of the study cohort background characteristics, while Figure 2 presents an example of the initial presentation and radiographic findings in severe ankle fractures that require talofibular trans-syndesmotic screws.

It was determined that the age of patients whose tibiofibular screw was removed followed a bimodal distribution, peaking in early adulthood and after 65 years of age. Patients in the “screw removal” group were significantly younger than patients in the control group of patients without screw removal, as 59.9% were in the 18–40 age group, compared to only 24.0% in the same age category of the control group (*p*-value < 0.001). Patient gender also followed different proportions between the two study groups. It was observed in the tibiofibular screw-removal group that 60.8% were men, compared with 45.8% in the non-removal group (*p*-value = 0.013). A proportion of 47.4% of patients in the entire cohort had a normal body mass index, and the remaining 41.5% were overweight. Substance-use behavior identified that 10.4%, and, respectively, 15.6% of patients from both groups, were frequently consuming alcohol, while a third of all the research cohort were smokers. Cardiovascular and metabolic diseases such as diabetes mellitus were the most common comorbidities in both study groups, without significant differences in proportions and without significantly high prevalence in a population. However, digestive and liver disease was a significant finding in the tibiofibular screw non-removal group, likely due to the older age of these patients and chronic alcohol consumption. 

Table 2 describes the orthopedic features and imaging characteristics of the studied patients. The side of the injury was proportionally equivalent between both groups, with a slightly higher prevalence of the right limb. The mean time between injury and definitive surgery was no longer than four days, while the fracture type was bimalleolar in 51.6% of all cases. The Danis–Weber classification distinguishes between three types of ankle fractures, where the most severe, type C, comes with tibiofibular diastasis; therefore, most of the patients included in the current study were classified as type C (93.8%). The remaining 6.2% were type B without diastasis that required screw fixation due to various reasons.

It was observed that trauma occurred significantly more often with high energy in the screw-removal patient group (60.4% vs. 46.9%, *p*-value = 0.026). ICU admissions were fairly low, although statistically significantly different between study groups, being, respectively, 7.1% in the first group and 14.6% in the second group (*p*-value = 0.036), and the average duration of stay in the ICU was 3.2 days in the first group, respectively, and 4.0 days in the screw non-removal group (*p*-value = 0.014). Similarly, the duration of first intervention hospitalization was longer for the second group, although the total duration of medical leave was significantly shorter for the patients whose tibiofibular screw was not removed, likely due to the absence of a secondary intervention to remove the screw. Therefore, the median duration of medical leave was 21 days in the first group, respectively, and 15 days in the second group (*p*-value < 0.001).

Short- and medium-term orthopedic complications after intervention were varied but did not affect a high number of patients, the most frequent being early degenerative joint disease, osteoarthritis, and wound infection, without significant difference in proportions between groups. Although there was a statistically significant difference in the proportions of patients with loss of reduction after orthopedic surgery between the two study groups (8.5% vs. 2.1%, *p*-value = 0.034), the difference likely happened due to our inclusion criteria. Therefore, those who had a loss of reduction had the screws removed for a second intervention, therefore being included in the first group of 212 patients with screw removal. The other two patients with loss of reduction from the second study group did not have their screws removed due to existing comorbidities contraindicating an intervention. 

Patient follow-up was performed six months after the initial intervention. The mean AOFAS score was significantly better in patients who had their tibiofibular screw removed in a secondary intervention. Although most patients from both groups underwent post-operative rehabilitation (64.9%), it was observed at the six-month follow-up that the average AOFAS score was significantly better in the group of patients with screw removal (92.6 vs. 88.4 out of a maximum of 100, *p*-value < 0.001), as presented in Table 3. These differences likely occurred due to age differences, since the second group was comprised of significantly older individuals. Furthermore, screw breakage was observed at the six-month follow-up in 18 patients (18.8%), although they did not show any significant consequences. 

The Lauge–Hansen classification showed that most patients in both groups suffered a supination external rotation type of fracture, although with significant differences in proportions (45.8% in the screw-removal group, vs. 33.3% in the non-removal group, *p*-value = 0.016). The range-of-motion assessment was also statistically significantly different in proportions, most of which showed excellent movement (45.3% vs. 34.4%, *p*-value = 0.038). Regarding the Van Dijk osteoarthritis scale, the current study did not identify any significant differences among the analyzed groups. The most prevalent score was 1 in both groups, with a slightly higher but insignificant proportion among patients with tibiofibular screw removal (59.4% vs. 49.0%). 

Lastly, radiographic changes were recorded at follow-up in both study groups, as observed in Table 4. It was observed that patients whose tibiofibular screw was not removed had a significantly smaller tibiofibular clear space (3.6 mm vs. 3.8 mm, *p*-value = 0.012), and, respectively, a lower TCS proportion change (7.3% vs. 15.6%, *p*-value = 0.045). Furthermore, the incisura fibularis change was significantly smaller in the screw non-removal group (9.4% vs. 19.3%, *p*-value = 0.028).

## 4. Discussion

### 4.1. Literature Findings

In accordance with our study, it was previously observed that there were no statistically significant changes when comparing measurements taken before and after the removal of the syndesmotic screw for the tibiofibular overlap (TOL) and the tibiofibular clear space (TCS) measures. When the measurements were compared after the syndesmotic screws were removed, the analysis revealed significant differences in regard to the tibiofibular overlap and tibiofibular clear space measurements, but not in regard to the medial clear space measurement [30].

In this particular research, we discovered that there was a decline in tibiofibular overlap at follow-up, whereas there was a rise in tibiofibular clear space in patients undergoing screw removal, suggesting that syndesmotic diastasis took place somewhere between the insertion of the syndesmotic screw and the completion of the last follow-up after the screw was removed. However, it is still unclear if diastasis occurred before the removal of the screws rather than after they were removed and follow-up was performed. These findings are in accordance with the findings of very recently published research [31]. Another study evaluated syndesmotic reduction using CT images taken two weeks after syndesmotic screw fixation and one year following screw removal, discovering that the anterior distance between the tibia and the fibula was substantially greater one year after the screws were removed.

Patients whose screws were not removed were compared to patients who underwent syndesmotic screw removal one year following syndesmotic screw fixation. Another study investigated this, reporting that there were no discernible differences between the two groups in TCS measurements [32]. It was described that when diastasis occurred, it was likely before the removal of the syndesmotic screws and that there were no substantial alterations to the syndesmotic space after the screws were removed. Another study conducted radiographic measurements following syndesmotic fixation and screw removal, reporting when diastasis was seen after the screw removal [33]. When the screws were removed, all patients were permitted to bear weight while wearing a boot that could be removed to prevent the restriction of ankle range of motion as well as to counter potential decreases in muscle power and post-operative functional outcomes. These results were confirmed in an older study stating that removing syndesmotic screws was essential when accompanied by weight-bearing activities before screw removal [34].

In spite of the high reported incidence of syndesmotic screw removal, the clinical and radiographic consequences of this procedure are not well-researched. As a result, there is a great deal of confusion about the surgical options that should be made for patients who are experiencing painful symptoms. Some findings suggest that removal one year after the first fixation is not related to recurrent diastasis while at the same time relieving the pain and discomfort associated with hardware [11]. Another option for syndesmotic fixation is a syndesmotic button that can benefit the patient by not having hardware removal, as in the case of screw fixation.

In the case of screw fixation, it has been hypothesized that recurrent diastasis may be caused by the early removal of hardware prior to the consolidation of syndesmotic ligaments, because it is unknown how long the syndesmotic ligament needs to heal [35]. This method of fixation makes it possible for the patient to continue exercising and bearing weight on the injured limb during what is believed to be the healing period without having to remove any of the hardware [36].

The financial burden that is associated with the care of syndesmotic injuries is another important concern, considering that screw removal increases the costs of managing a particular patient because of the need for a second surgical intervention. On the other hand, when screws are retained, the procedure is considered cost-effective only if the rate of subsequent operations is kept at or below 17.5% of all cases. However, a recent meta-analysis compared dynamic screw fixation with permanent screw fixation and found that dynamic screw fixation was associated with roughly four times as many implant-related reoperations as permanent screw fixation [37].

Even though static fixation is more often used, the biomechanical properties of foot movements and functions may help to explain why dynamic fixation is clinically more beneficial than static fixation, as several studies suggest [38]. It is possible that a more rapid healing process and clinical recovery may have been achieved with the help of dynamic devices that allowed for the restoration of more physiological motions of the syndesmosis. As a consequence, a shorter amount of time to return to work and perform physical activity was found in several studies [39]. Although our research did not include patients with dynamic fixation of the ankle, data suggest a significantly quicker return to work when the tibiofibular screw was not removed, allowing for the quicker mobilization of the patient. Another potential benefit of dynamic fixation, particularly when using suture buttons, is that it could make anatomic reduction simpler to achieve by allowing for greater mobility and better self-centring of the syndesmosis [40].

Regarding the complications observed after surgery, researchers suggest that lesions of the ankle syndesmosis may lead to substantial disability and longer medical leave, as identified in our study. The early detection and effective treatment of complications are key to restoring stability, mobility, and strength, with the ultimate goal of recovering the function of the ankle that existed before the injury [41]. Otherwise, patients will show persistent pain, slow recovery, repeated ankle sprains, heterotopic ossification, and ankle instability, many of these being difficult to diagnose, and improving outcomes from these complex injuries requires awareness of the mechanism of injury. A secondary surgical intervention, such as the insertion of a syndesmotic screw or a suture button, is required in order to address the instability of the syndesmosis as a complication. A distal tibiofibular rotational bone–plug fusion is the recommended course of treatment for injuries that have been present for more than six months, while an ankle fusion should be explored for patients who have developed osteoarthritic abnormalities in the ankle [42].

### 4.2. Study Limitations and Future Perspectives

The current study successfully met the participants’ inclusion criteria and sample size requirements for statistical significance, however, the convenience sampling technique for the control group may not be entirely representative of the overall population, hence raising the possibility of selection bias. Overall, several limitations exist. First, the retrospective type of study impacts our results as the research depends on the accuracy of both patient information tracking and the digital transcription of data from paper records. As a retrospective study, the degree of evidence is weaker compared to prospective studies, as well as increasing the risk for misclassification and confounding bias, and erroneous data about temporal relationships between study variables. Furthermore, different types of tibiofibular plates and screws were used depending on the patient’s anatomy and availability of materials; therefore, different results at the six-month follow-up might occur. The current study also included open fractures, which might affect the outcomes due to a higher level of trauma.

## 5. Conclusions

The current study did not identify many significant differences between patients with tibiofibular syndesmotic screw removal and those whose screw was not removed. The most significant orthopedic findings were a loss of reduction occurring more often when the tibiofibular screw was removed, as well as observing more significant radiographic changes in the same group regarding the tibiofibular clear space and incisura fibularis change. It seems that the costs of treatment are significantly reduced if a secondary intervention for screw removal is avoided, as well as a shorter medical leave for those who undergo a single intervention.

## Figures and Tables

**Table 1:** Comparison of the study cohort background characteristics.

Variables	Tibiofibular Screw Removal (*n* = 212)	Tibiofibular Screw Non-Removal (*n* = 96)	*p*-Value *
Age			<0.001
18–40 years	127 (59.9%)	23 (24.0%)	
40–65 years	31 (14.6%)	24 (25.0%)	
>65 years	54 (25.5%)	49 (51.0%)	
Sex			0.013
Men	129 (60.8%)	44 (45.8%)	
Women	83 (39.2%)	52 (54.2%)	
BMI **			0.107
Underweight (<18.5 kg/m^2^)	21 (9.9%)	13 (13.5%)	
Normal weight (18.5–25.0 kg/m^2^)	109 (51.4%)	37 (38.5%)	
Overweight (>25.0 kg/m^2^)	82 (38.7%)	46 (47.9%)	
Area of residence (urban)	133 (62.7%)	52 (54.2%)	0.154
Relationship status (married)	141 (66.5%)	70 (72.9%)	0.262
Level of income (average or higher)	122 (57.5%)	56 (58.3%)	0.897
Level of education (higher education)	151 (71.2%)	59 (61.5%)	0.088
Occupation (employed)	116 (54.7%)	60 (62.5%)	0.201
Substance-use behavior			
Frequent alcohol consumption	22 (10.4%)	15 (15.6%)	0.189
Frequent smoker	58 (27.4%)	33 (34.4%)	0.211
Use of drugs	9 (4.2%)	2 (2.1%)	0.343
Chronic comorbidities			
Cardiac	37 (17.5%)	22 (22.9%)	0.259
Lung	15 (7.1%)	13 (13.5%)	0.067
Metabolic	10 (10.4%)	16 (16.7%)	0.120
Cerebrovascular	5 (2.4%)	6 (6.3%)	0.088
Digestive and liver	5 (2.4%)	7 (7.3%)	0.038
Kidney disease	7 (3.3%)	7 (7.3%)	0.119
Cancer	11 (7.0%)	5 (9.3%)	0.589
Depression	2 (0.9%)	4 (4.2%)	0.057
Other	7 (3.3%)	8 (8.3%)	0.057

* Chi-square or Fisher’s exact test; ** Weight measured when pregnancy loss occurred; BMI—Body mass index.

**Table 2:** Orthopedic features and imaging characteristics.

Variables	Tibiofibular Screw Removal (*n* = 212)	Tibiofibular Screw Non-Removal (*n* = 96)	*p*-Value *
Poor soft tissue condition	28 (13.2%)	22 (22.9%)	0.032
Side of injury			0.232
Left	84 (39.6%)	45 (46.9%)	
Right	128 (60.4%)	51 (53.1%)	
Time between injury and definitive surgery (mean ± SD)	3.6 ± 2.2	4.1 ± 2.5	0.079
Danis–Weber classification			0.637
B	14 (6.6%)	5 (5.2%)	
C	198 (93.4%)	91 (94.8%)	
Fracture type			0.411
Unimalleolar	54 (25.5%)	23 (24.0%)	
Bimalleolar	113 (53.3%)	46 (47.9%)	
Trimalleolar	45 (21.2%)	27 (28.1%)	
Trauma type			0.026
Low-energy	84 (39.6%)	51 (53.1%)	
High-energy	128 (60.4%)	45 (46.9%)	
Outcomes			
ICU admission	15 (7.1%)	14 (14.6%)	0.036
Days in the ICU (mean ± SD)	3.2 ± 2.5	4.0 ± 2.9	0.014
First intervention hospitalization (mean ± SD)	5.3 ± 2.0	6.8 ± 3.1	0.001
Second intervention hospitalization (median, IQR)	2 (0–2)	–	–
The total duration of medical leave (median, IQR)	21 (15–27)	15 (6–20)	<0.001
Orthopedic complications			
Loss of reduction	18 (8.5%)	2 (2.1%)	0.034
Wound infection	17 (8.0%)	9 (9.4%)	0.691
Hemarthrosis	11 (5.2%)	3 (3.1%)	0.420
Osteomyelitis	3 (1.4%)	1 (1.0%)	0.788
Osteoarthritis	19 (9.0%)	10 (10.4%)	0.685
Early degenerative joint disease	23 (10.8%)	9 (9.4%)	0.694
Skin necrosis	4 (1.9%)	1 (1.0%)	0.864
Neuroparalysis	2 (0.9%)	0 (0.0%)	0.339
Sepsis	6 (2.8%)	2 (2.1%)	0.702
Venous thrombosis	8 (3.8%)	5 (5.2%)	0.561

* Chi-square or Fisher’s exact test; ICU—Intensive care unit; SD—Standard Deviation; IQR—Interquartile Range.

**Table 3:** Patient follow-up at six months after the initial surgery.

Variables	Tibiofibular Screw Removal (*n* = 212)	Tibiofibular Screw Non-Removal (*n* = 96)	*p*-Value *
Average time to screw removal (days)	63.1 ± 7.7	–	–
Screw breakage	–	18 (18.8%)	–
Post-operative rehabilitation			0.668
Yes	136 (64.2%)	64 (66.7%)	
No	76 (35.8%)	32 (33.3%)	
AOFAS score (mean ± SD)	92.6 ± 6.1	88.4 ± 5.8	<0.001
Lauge–Hansen classification			0.016
SER	97 (45.8%)	32 (33.3%)	
PER	68 (32.1%)	27 (28.1%)	
SA	22 (10.4%)	21 (21.9%)	
PA	25 (11.8%)	16 (16.7%)	
Van Dijk osteoarthritis scale			0.389
0	42 (19.8%)	25 (26.0%)	
1	126 (59.4%)	47 (49.0%)	
2	37 (17.5%)	20 (20.8%)	
3	7 (3.3%)	4 (4.2%)	
ROM assessment			0.038
Poor	21 (9.9%)	18 (18.8%)	
Good	95 (44.8%)	46 (47.9%)	
Excellent	96 (45.3%)	33 (34.4%)	
Quality of reduction			0.258
Poor	17 (8.0%)	13 (13.5%)	
Good	88 (41.5%)	41 (42.7%)	
Excellent	107 (50.5%)	42 (43.8%)	
Estimated costs (EUR), median (IQR)	900 (400–1200)	400 (200–800)	<0.001

* Chi-square or Fisher’s exact test; ROM—range of motion; SER—supination external rotation fracture; PER—pronation external rotation fracture; SA—supination adduction; PA—pronation abduction; AOFAS—The American Orthopedic Foot and Ankle Society; IQR—interquartile range.

**Table 4:** Differences in radiographic changes between two follow-ups in the study groups.

Variables (Mean ± SD)	Tibiofibular Screw Removal (*n* = 212)	Tibiofibular Screw Non-Removal (*n* = 96)	*p*-Value *
TOL	8.8 ± 1.7	8.4 ± 2.6	0.109
TCS	3.8 ± 1.0	3.6 ± 0.9	0.012
IFD	4.3 ± 0.8	4.1 ± 1.2	0.085
TOL change	18.4%	11.5%	0.126
TCS change	15.6%	7.3%	0.045
IFD change	19.3%	9.4%	0.028

* Student’s *t*-test; TCS—tibiofibular clear space; TOL—tibiofibular overlap; IFD—incisura fibularis depth; SD—standard deviation.

## Data Availability

The data presented in this study are available on request from the corresponding author.
